# INDI—integrated nanobody database for immunoinformatics

**DOI:** 10.1093/nar/gkab1021

**Published:** 2021-11-08

**Authors:** Piotr Deszyński, Jakub Młokosiewicz, Adam Volanakis, Igor Jaszczyszyn, Natalie Castellana, Stefano Bonissone, Rajkumar Ganesan, Konrad Krawczyk

**Affiliations:** NaturalAntibody, Al. Piastów 22, Szczecin, Poland; NaturalAntibody, Al. Piastów 22, Szczecin, Poland; Harvard Medical School, 240 Longwood Ave, Boston, MA, USA; NaturalAntibody, Al. Piastów 22, Szczecin, Poland; Abterra Biosciences Inc. 3030 Bunker Hill Street Suite 218, San Diego, CA 92109, USA; Abterra Biosciences Inc. 3030 Bunker Hill Street Suite 218, San Diego, CA 92109, USA; Alector, South San Francisco, CA 94080, USA; NaturalAntibody, Al. Piastów 22, Szczecin, Poland

## Abstract

Nanobodies, a subclass of antibodies found in camelids, are versatile molecular binding scaffolds composed of a single polypeptide chain. The small size of nanobodies bestows multiple therapeutic advantages (stability, tumor penetration) with the first therapeutic approval in 2018 cementing the clinical viability of this format. Structured data and sequence information of nanobodies will enable the accelerated clinical development of nanobody-based therapeutics. Though the nanobody sequence and structure data are deposited in the public domain at an accelerating pace, the heterogeneity of sources and lack of standardization hampers reliable harvesting of nanobody information. We address this issue by creating the Integrated Database of Nanobodies for Immunoinformatics (INDI, http://naturalantibody.com/nanobodies). INDI collates nanobodies from all the major public outlets of biological sequences: patents, GenBank, next-generation sequencing repositories, structures and scientific publications. We equip INDI with powerful nanobody-specific sequence and text search facilitating access to >11 million nanobody sequences. INDI should facilitate development of novel nanobody-specific computational protocols helping to deliver on the therapeutic promise of this drug format.

## INTRODUCTION

Antibodies are proteins capable of recognizing a specific molecular site on a potentially noxious molecule (antigen), starting an immune response (1). Because of their binding malleability they are the primary class of biotherapeutics (6 of 10 blockbusters and market worth ∼100b$). Clinical development of an antibody-based drug is complex and arduous, often taking years (2,3). The difficulties stem from the complexity of antibodies: they are composed of two polypeptide chains which need to be co-engineered and co-expressed. The protein itself is large which makes delivery difficult especially in challenging cases such as tumor penetration. Therefore, there is a lot of interest in exploring alternative antibody formats with more favorable therapeutic properties. One of these is a subclass of antibodies discovered in camelids - the nanobody (alternatively called the single domain antibody or VHH) (4).

Nanobodies bear similarity to normal antibodies however their antigen binding region is composed of just one polypeptide chain. Nanobodies retain molecular recognition advantages of antibodies and exhibit improved biophysical and therapeutic properties as a result of their smaller size (5). Nanobodies are reported to be more stable, soluble and able to recognize cryptic epitopes and penetrate tissues inaccessible to normal antibodies (4,6). The interest in this direction is reflected by multiple novel nanobodies in either regulatory filing or in the late clinical-trial stages (7) and an increasing volume of patents reporting nanobody sequences (8). In 2018 the first nanobody drug was approved (Caplacizumab (9), by Ablynx), confirming the therapeutic viability of such molecules. Developing nanobodies using traditional laboratory approaches will still require years before they reach the clinic. Computational approaches could accelerate this process, delivering life-saving therapeutics faster and make them more affordable.

Computational methods to design antibodies are already mature enough to provide value in monoclonal antibody therapeutic pipelines (10). By contrast, though nanobodies were discovered close to 30 years ago (11), they attracted less attention in collating data and developing computational protocols addressing these molecules (10). Development of approaches enabling computational design of nanobodies rely on ever deeper analysis of their sequence diversity (12,13) structural conformations (14), antigen-binding preferences (15), attempts at modifying their binding mode (16) and emerging deep-learning methods tackling this format (17).

Successful computational protocols addressing nanobodies rely on sound sequence and structure data describing the biology of these molecules. A pioneering effort in this direction was achieved by the iCAN (18) and sdAB-DB (19) databases that to our knowledge were first attempts at collection of nanobody-related data. These databases focused on manual identification of antibodies. As a result, they hold a relatively small number of publicly available nanobody data, with sd-AB reporting 1452 sequences and iCAN 2391. Data collection frameworks need to keep up pace with the ever-increasing amount of biological sequence data in the public domain. To tackle this, we created INDI- Integrated Nanobody Database for Immunoinformatics. INDI is a novel nanobody database that collates nanobody information from all major data repositories in the public domain, chiefly in automated fashion.

## DATA COLLECTION

We identified five major sources of biological sequence information: NCBI GenBank (20), Protein Data Bank (21), patents (8), next-generation sequencing (NGS) repositories (22,23) and scientific publications. These sources provide a good coverage associated with systematic repositories collecting protein information from scientific literature and patent documents.

Because of the heterogeneity of the sources, we take the variable sequence of the nanobody as the common denominator between the datasets. Though in many cases, especially in scientific publications, only CDR-H3 sequences are published, we decided to exclude such data from INDI. This choice was taken as rational nanobody engineering requires the entire variable region context for modeling endeavors such as humanization (24) or structural modeling (25). We require the nanobody sequences to have all three Complementarity Determining Regions (CDRs) present and only contain 20 canonical amino acids. Sequences are linked with metadata specific for the source dataset (Table 1).

**Table 1.** Contents of INDI in May 2021

Source	Unique sequences	Unique accessions	Main source	Metadata
Structures	535	804 PDB codes	Protein Data Bank (21)	PDB code, PDB title, authors, resolution technique, text headers of chain fasta files
NCBI GenBank	1858	2070 GenBank ids	GenBank (20)	GenBank ID, GenBank description/definition, reported organism, date, reference title reference link/pmid, reference journal, reference authors
Next-generation sequencing	11 228 600	Seven Bioproject ids	Sequence Read Archive (23)	BioProject id, SRA id
Patents	14 376	687 patent families	Patented Antibody Database (8)	Patent number, applicants, patent title, patent abstract
Manual	1268	109 papers	Scientific publications	Publication title, publication abstract, publication link

Data in INDI is divided into five distinct sources. For each source we provide the reference to the online resource we obtained the data from (with the exception of scientific publications), metadata associated with accessions in source as well as August 2021 statistics of the number of nanobodies we extracted.

**Figure 1.**
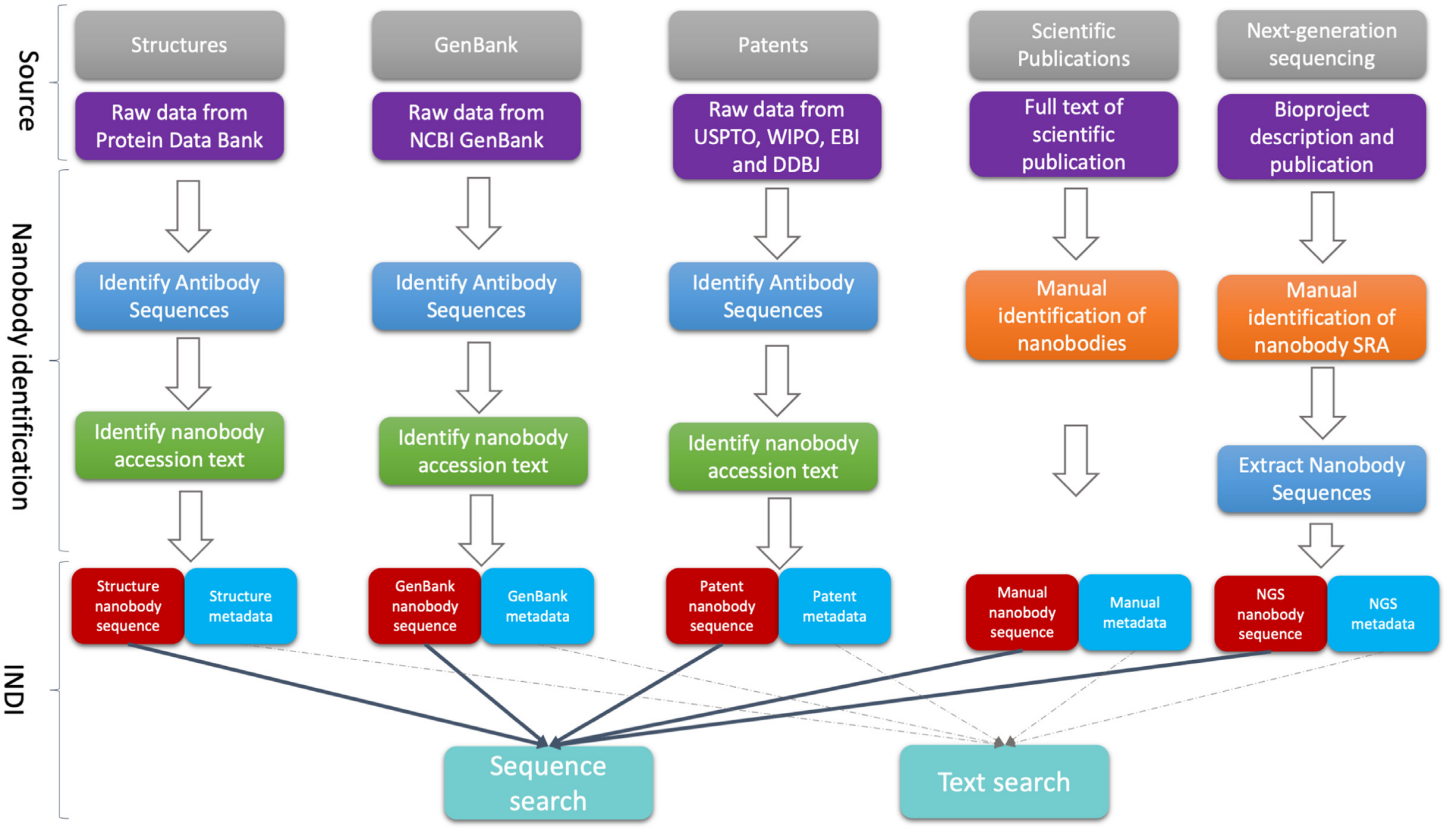
Data sources and information organization in INDI. We obtain nanobody data from five distinct sources: structures, GenBank, patents, scientific publications and NGS. Structures, GenBank and patents are suitable for automated identification divided into identification of antibody sequences and subsequent filtering of nanobody sequences based on text. Scientific publications and NGS are not suitable for automated identification and they require ad-hoc manual curation. Data from all sources are standardized into sequence and metadata indexes. The web-utility of INDI enables users to query nanobody sequence and metadata indexes spanning all five repositories.

Three of the datasets (PDB, GenBank and patents) are suitable for automatic curation of the data (Figure 1). Here, sequence entries are firstly analyzed for presence of antibodies employing Hidden Markov Models trained on antibody germline genes (26). Sequences where antibodies are detected, are further filtered for presence of nanobodies and thus inclusion in INDI. Accessions containing nanobodies are identified by natural language processing. Through analysis of nanobody-related keywords used in previous studies and our own iterative analysis of nanobody accessions we created a set of keywords relating to nanobodies: *vhh*, *nanobody*, *single domain antibody*, *domain antibody*, *single variable domain*. Arbitrary pieces of text from our heterogenous sources were normalized by case-folding and stemming and then checked for inclusion of the said keywords.

NGS and scientific papers are curated manually because of limited standardization. Though NGS depositions are increasingly standardized as a result of the AIRR community efforts (27), specific formats such as nanobodies need to be treated in an ad-hoc manner. For instance, NGS study descriptions need to be manually checked for inclusion of nanobodies so as to avoid errors where single domain antibodies are deposited alongside canonical antibodies (12). Scientific publications containing nanobodies are identified an ad-hoc manner and nanobody sequences found by a human curator included in INDI as part of the ‘manual’ dataset.

In August 2021, INDI held more than 11 million nanobody sequences spanning our five data sources (Table 1). Contents and specific data collection strategies for each of the five datasets making up INDI are described below.

### NCBI GenBank

We collected the protein sequences from the ftp facility of NCBI GenBank in February 2021. Protein sequences were identified as translation entries associated with each GenBank entry. We discarded sequences that were either too short (<70 amino acids long) or too long (>600 amino acids long), with both numbers chosen to capture the lengths of the nanobody variable regions. In total, 16 271 610 entries matched these criteria. Antibody sequences were identified employing our adapted version of antibody numbering software (8,26).

Individual GenBank entries were identified as containing nanobodies if their textual description contained a set of *nanobody-specific keywords* (19,28) and if the declared organism matched one of: ‘Lama glama’, ‘Camelus dromedarius’, ‘Vicugna pacos’, ‘synthetic construct’, ‘Camelus bactrianus’, ‘Camelidae’ or ‘unidentified unclassified sequences’. Employing this protocol, in August 2021, we identified a total of 1,858 unique variable nanobody sequences from a total of 2070 GenBank accessions.

Each sequence from GenBank is associated with text metadata specific to this repository. These entries were GenBank ID, GenBank description/definition, reported organism, date, reference title, reference link/pmid, reference journal and reference authors.

### Patents

We extracted patent nanobody sequences from our Patented Antibody Database (8) in February 2021. Patent families containing nanobodies were identified by virtue of their classifications C07K2317/569 (Single domain, e.g. dAb, sdAb, VHH, VNAR or nanobody®), C07K2317/22 (from camelids, e.g. camel, llama or dromedary) or if the title and abstract contained one *nanobody-specific keywords*. A Total of 1013 patent families satisfied the nanobody keyword or category requirements.

In certain cases, claims are laid not only to nanobody sequences within a patent document but also to canonical antibodies. Such families are characterized by a mix of heavy chains aligning to camelid and non-camelid germlines and presence of light chains. Total of 354 families contained both nanobodies and heavy chains of canonical antibodies. Without manual curation it was impossible to tell whether the individual non-camelid heavy chains could function as antibodies on their own. So as to maximize the precision of automated identification, in INDI we only retain sequences where the document declares presence of nanobodies and we only detect heavy chain sequences aligning to camelid germline genes. This resulted in a total of 14 376 unique variable region sequences from 687 patent families.

Each nanobody patent sequence is associated with metadata from the original document. These are patent number, the applicants (e.g. company), patent title and abstract.

### Structures

We sourced nanobody structures from the Protein Data Bank (PDB) ftp facility (21). Antibody sequences from the PDB were identified according to the protocol of the Structural Antibody Database (SAbDab (29)). We excluded sequences that had noncanonical amino acids in their sequences (e.g. 1I3U, 6ULF, 6ANA or 6VLN). From such a constrained set of antibodies, structures containing nanobodies were identified by inclusion of *nanobody-specific keywords* in text associated with the PDB, specifically descriptions of the chains.

Such targeted approach was necessary to distinguish between cases where canonical antibodies might be present alongside nanobodies (e.g. 6ZCZ, 7JOO). Furthermore, it allows us to weed out cases such as 6QKD that reports a VHH-based antibody or 4O9H, which is a camelid Fab rather than a nanobody. Here we also detect human-only single domain antibodies such as 5N88. In May 2021, this extraction approach identified 535 unique nanobody chains from 804 PDBs.

Each structural nanobody entry is linked to metadata originating from the PDB accession. These entries are the PDB code, accession title, authors, resolution, technique (e.g. X-ray) and text headers of FASTA files associated with individual chains.

### Next generation sequencing

We identified eight bioprojects reporting next-generation sequencing of nanobodies by employing the text-search utility at NCBI: PRJDB2382, PRJEB7678, PRJNA642677, PRJDB7792, PRJEB25673, PRJNA516512, PRJNA638614 and PRJNA321369. We extracted the nanobody sequences contained within the bioprojects as described previously (22). In brief, SRA files containing raw reads are analyzed using IgBlast (30) that translates the nucleotide sequences into amino acids. Sequences that are free of stop codons and that contain all three CDRs are retained.

In the case of the Bactrian camel study that contained samples of both nanobodies and canonical antibodies, we only made the VHH samples part of INDI that corresponded to SRAs SRR3544218, SRR3544220 and SRR3544222. Though, PRJNA516512 advertised presence of nanobodies, the resulting IgBlast processed sequences did not satisfy our inclusion criteria due to incompleteness of chains. The remaining seven bioprojects contributed a total of 11 228 600 unique variable region sequences. Each NGS nanobody sequence is associated with the SRA file it originated from and metadata of the Bioproject.

### Manual curation

In certain cases individual nanobody sequences that are reported in publications are not deposited in systematic repositories such as GenBank or the PDB. Such sequences are typically contained in supplementary materials. The reporting is not standardized and therefore challenging for automated approaches. Therefore, we created a ‘manual’ category for all sequences that are obtained by human curation of sequences originating directly from scientific publications. Because of lack of automated means, data in this database will be updated in an ad-hoc way. In each case, the variable region sequences of nanobodies are manually identified by a human curator and automatically filtered for presence of all three CDRs and lack of non-canonical amino acids. The variable region sequences are associated with the metadata of the original publication in the form of its title and abstract.

### Content analysis

We analyzed the contents of our database to offer an overview of nanobody-specific features as reported in heterogenous sources integrated within INDI.

Nanobodies are known to differ from canonical heavy chains by presence of hallmark residues in framework region 2 positioned on the VL interface in canonical antibodies (31). Hydrophilic character of certain hallmark motifs is thought to increase the solubility on the interfaces exposed by lack of the light chain. Hallmark residues are IMGT positions 42, 49, 50 and 52. We analyzed the amount of hallmark motifs across the heterogeneous datasets and in sdab-DB (Table 2).

**Table 2.** The most common hallmark residue motifs across five INDI datasets and sdab-DB. We calculated the statistics of combination of amino acids in IMGT positions 42, 49, 50 and 52 in all INDI datasets as well as sdab-DB

Manual (126 motifs)		Structures (83 motifs)		Patents (452 motifs)		NGS (12,307 motifs)		GenBank (206)		sdab-DB (152)	
Motif	% total	Motif	% total	Motif	% total	Motif	% total	Motif	% total	Motif	% total
FERF	24%	FERF	27%	FERF	30%	FERG	30%	FERF	20%	FERF	33%
FERG	16%	FERG	15%	YQRL	15%	VGLW	10%	FERG	17%	FERG	14%
YERW	10%	YQRL	9%	FERG	11%	YQRL	9%	VGLW	13%	YQRL	8%
VGLW	5%	VGLW	6%	VGLW	4%	FERF	9%	YQRL	7%	VGLW	8%
YQRL	5%	YERL	4%	FERL	2%	FERA	2%	YERL	3%	YERL	4%
YERL	2%	YERW	3%	YKRL	1%	YERL	2%	YERF	2%	FERA	2%
YERF	1%	FERW	1%	YERL	1%	FGRG	1%	IGLW	1%	YERW	1%
YQRW	1%	FERA	1%	FGRF	1%	FERE	1%	FERA	1%	YERF	1%
FERA	1%	YERG	1%	YQRF	1%	FARG	1%	FERL	1%	FERL	1%
WQRL	1%	YERF	1%	IGLW	1%	FERR	1%	FERV	1%	YQRW	1%

The percentage of each motif is given with respect to the total number of sequences in any given source.

We note a large number of possible hallmarks in each dataset that is dominated by several motifs. The most common motifs are FERF, FERG, VGLW and YQRL. The FERF motif is typical of more soluble nanobodies (32). The VGLW motif resembles human heavy chains but can be obtained from native llama repertoires (32). Altogether, the only outlier dataset considering abundance of motifs is the most sequence-abundant dataset: NGS. Here the most common motif across all other sources (FERF, with least frequency of 20%) occurs only in 9% of sequences, with FERG motif dominating. We checked whether this could be an effect of a single NGS study biasing the statistic and we plotted the top 10 motifs for each NGS study in Table 3. We note that FERG motif is indeed the most common in five out of seven NGS studies. The only obvious outlier is bioproject PRJDB2382, where top motifs do not find correspondence with any other source. This could be an effect of biasing the library by immunization with IZUMO1. Altogether, discrepancies between the NGS datasets and other sources in INDI shows that there is a difference in top hallmark motifs as seen from sources containing greater number of artificially developed sequences (e.g. structures, patents, GenBank) and naturally-sourced ones (NGS). This speaks in favor of informed selection of the data from a specific source so as not to bias immunoinforamtics methods.

**Table 3.** The most common hallmark residue motifs across the seven NGS datasets in INDI

PRJDB7792 (869)		PRJNA638614 (6061)		PRJNA321369 (5722)		PRJEB7678 (4525)		PRJNA642677 (5478)		PRJEB25673 (1423)		PRJDB2382 (1984)	
Motif	%Total	Motif	%Total	Motif	%Total	Motif	%Total	Motif	%Total	Motif	%Total	Motif	%Total
FERG	46%	FERG	37%	FERG	32%	YQRL	26%	FERG	33%	FERG	44%	VALW	27%
FERA	8%	FERA	5%	VGLW	21%	FERG	17%	FERF	13%	VGLW	14%	YQRL	16%
FKRG	5%	VGLW	5%	FERA	3%	FERF	15%	VGLW	8%	YERW	6%	YERL	13%
YQRL	4%	FERF	4%	YECL	1%	VGLW	9%	YQRL	8%	FERA	5%	VGLW	3%
FGRG	2%	YERL	4%	FGLW	1%	YERL	2%	FERA	2%	FERF	3%	FERG	2%
FQRG	2%	FARG	3%	FGRG	1%	FQRL	2%	YERL	1%	AGLW	2%	FERF	1%
SERG	1%	FERE	2%	FERR	1%	VGPW	1%	FGRG	1%	FERE	2%	YQRM	1%
YERG	1%	FERW	1%	FARG	1%	FERL	1%	FERR	1%	FKRG	1%	YQRV	1%
VERG	1%	FGRG	1%	FERE	1%	FERW	1%	FERE	1%	VERG	1%	YQRW	1%
FDRG	1%	VGPW	1%	FQRG	1%	YERG	1%	FERV	0%	VGPW	1%	YQRF	1%

We calculated the statistics of combination of amino acids in IMGT positions 42, 49, 50 and 52 in the seven INDI NGS bioprojects. The percentage of each motif is given with respect to the total number of sequences in any given bioproject.

To offer a more granular view of the relationship between sequences in the five sources in INDI, we performed a clustering analysis. We used CD-HIT to cluster all the sequences we can find in INDI at 70% sequence identity, to reveal broad-brush overlaps between all the datasets. Clustering all INDI sequences at 70% sequence identity resulted in 75 384 clusters. Most of the sequences fall into a smaller (relative to 75,384) number of clusters (Table 4). The top 8, 41, 227 and 1148 clusters with most sequences, respectively account for 20%, 40%, 60% and 80% of all sequences in INDI. Majority of remaining clusters is composed of singletons originating from NGS datasets.

**Table 4.** Number of clusters and sequences falling within the same clusters at 70% sequence identity

#Sources in clusters	Clusters	Sequences
1	73 479	3 702 072
2	1245	1 275 004
3	410	914 843
4	161	1 050 308
5	89	4 303 563

For each cluster we noted the number of sources that contributed sequences – with the maximum number being five (NGS, patents, GenBank, structures and manual).

Because of the lack of size balance between NGS datasets with 11 228 600 sequences and other components of INDI with 18,037 sequences, we clustered the four remaining components, patent, manual, GenBank and structural sequences separately. Clustering was performed using CD-HIT at 70%, 80%, 90% and 99% sequence identity to stratify fine sequence differences between the datasets with results in Table 5. For each clustering, we noted the percentage of all sequences from a given source that were found in clusters with each other source. For instance in Table 5 at 70% CD-HIT cutoff, 89% of nanobody sequences from structures are found in clusters with nanobody sequences that were manually curated. Table 5 indicates that at sequence cutoffs of 70% and 80% where framework plays a dominant role, majority of sequences from four sources overlap with each other. At sequences identities of 90 and 99% which require high level of sequence identity both in framework and paratope, the overlaps are chiefly single digit percentages.

**Table 5.** Clustering analysis of non-NGS components of INDI

		Manual		Structure		GenBank		Patent	
CD-HIT sequence identity cutoff	Database	%Row sequences	%Column sequences	%Row sequences	%Column sequences	%Row sequences	%Column sequences	%Row sequences	%Column sequences
70	Manual	-	-	79	89	86	77	94	83
	Structure	89	79	-	-	93	71	95	76
	GenBank	77	86	71	93	-	-	93	85
	Patent	83	94	76	95	85	93	-	-
80	Manual	-	-	44	49	34	29	48	33
	Structure	49	44	-	-	53	28	69	34
	GenBank	29	34	28	53	-	-	69	40
	Patent	33	48	34	69	40	69	-	-
90	Manual	-	-	11	9	6	4	14	1
	Structure	9	11	-	-	16	4	32	4
	GenBank	4	6	4	16	-	-	49	8
	Patent	1	14	4	32	8	49	-	-
99	Manual	-	-	2	5	4	2	8	0
	Structure	5	2	-	-	11	3	27	1
	GenBank	2	4	3	11	-	-	38	6
	Patent	0	8	1	27	6	38	-	-

We clustered nanobody sequences from manual curation, structures, patents and GenBank using CD-HIT at 70%, 80%, 90% and 99% sequence identity. For each clustering cutoff we indicate the percentage of sequences from any given source that were clustered together with any sequences from another source. For instance, at clustering cutoff 80%, 49% of sequences from structures cluster with manually curated sequences.

**Figure 2.**
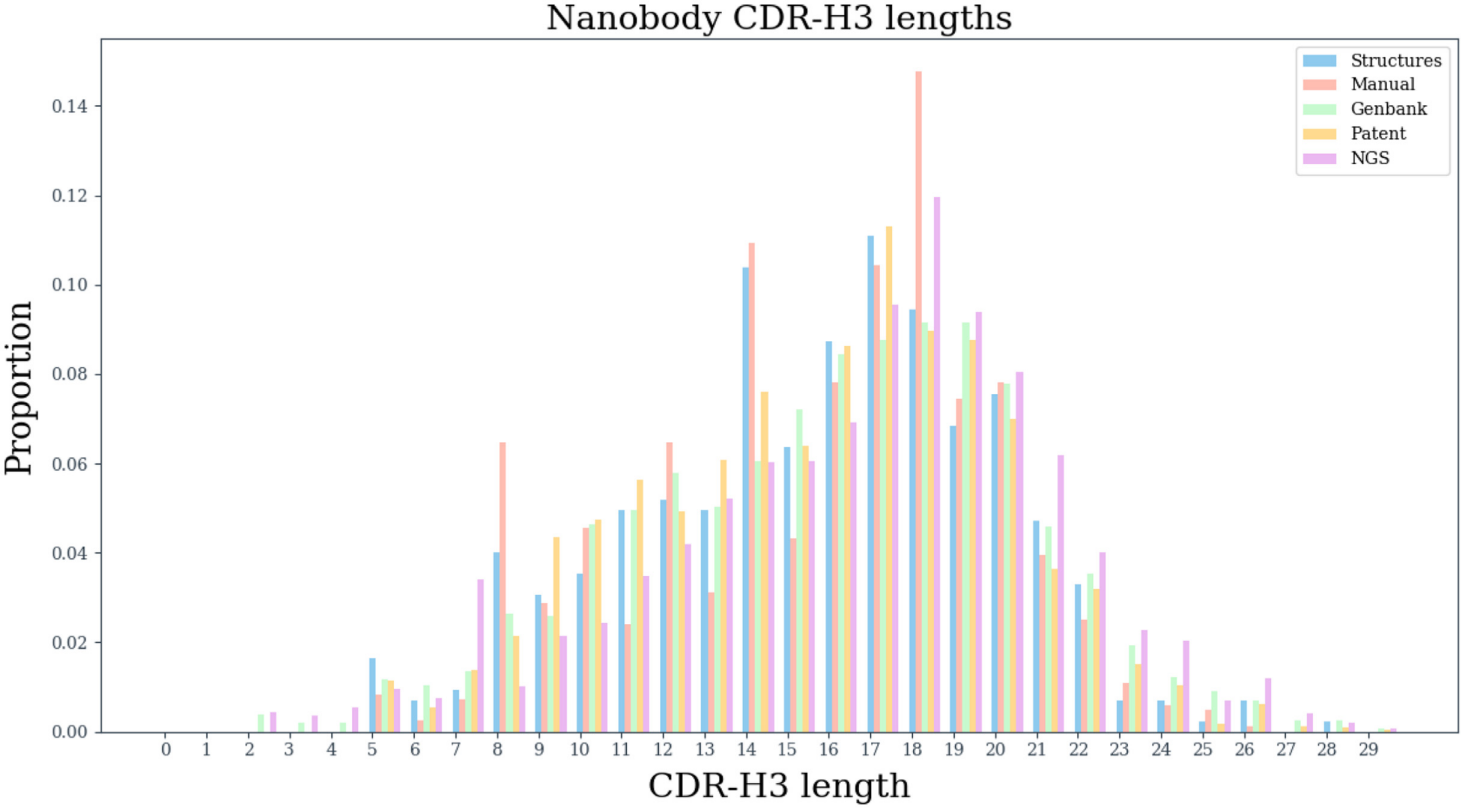
Distribution of IMGT CDR-H3 lengths in INDI. We extracted unique IMGT-defined CDR-H3 sequences from each dataset in INDI and noted their lengths.

CDR-H3 is the most variable portion of the paratope, providing the biggest differentiating factor between immunoglobulins. In nanobodies particularly, CDR-H3 is longer than in normal antibodies to account for the lack of the light chain (12,14,15). We plotted the IMGT-defined CDR-H3 distribution across five of INDI sources in Figure 2. The most common length across GenBank, manual and NGS datasets is 18 and in patents and structures 17 in line with previous estimates (13). The smoothest distribution can be attributed to NGS sequences that are the most voluminous. Certain sequences in our datasets are based on canonical antibodies that were produced without the light chain, reflected in a large number of manual and structural CDR-H3s with length of 14.

Altogether, our contrast of the five sources of sequences in INDI indicates previously known features associated with nanobodies, such as hallmark motifs or distinct CDR-H3 distribution. There are distinctions between the datasets as indicated by our clustering which speaks in favor of careful selection of correct datasets for immunoinformatic analyses.

### Comparison to other databases

In order to ensure that we capture nanobody information reliably, we performed a contrast to other resources that collect these molecules. We compared entries from INDI to those in sdAB-DB (20), structural nanobody entries curated by the Structural Antibody Database (SAbDab) (29), and the CoV-AbDab that curates some COVID-19-related nanobodies alongside canonical antibodies (33).

We checked whether all the PDB codes that we identified as nanobodies have the same annotation in SAbDab. All of the February 10th version of SAbDab nanobody-annotated sequences were found in INDI. The only exceptions were PDB codes that were subsequently changed in the PDB (e.g. 6h7k→ 6ibl, 6csy→6mxt). Entries from CoV-AbDab (17th March version) and sdAB-DB that were manually extracted from the literature were not found in INDI. All the entries that could be obtained by automatic means from the PDB or GenBank were shared between INDI, CoV-AbDab and sdAB-DB.

Entries from sdAB-DB that could have been obtained by automatic means but were not present in our database could be divided between light chain single-domain antibodies (e.g. sdAB-DB sdAb_2370_Sy, GenBank AAG49009, AAG49011) or single chain Fv (scFv) fragments that were deposited as separate chains (e.g. sdAb_1934_Sy, GenBank AKR15657). Certain entries that were present in sdAB-DB contained non-canonical amino acids which we do not include in INDI (e.g. sdAb_1339_Lg CAH60929). All the literature entries that were not found in INDI were subsequently entered – because of the comparison to CoV-AbDab, 18 of 109 manually documents and 384 of 1268 manually curated sequences are related to coronavirus.

We further compared the contents of INDI to sdAB-DB and iCAN by means of clustering. We employed CD-HIT (34) to cluster non-NGS and non-manual sequences in INDI together with these from sdAB-DB and iCAN. Clustering was performed at four levels of sequence identity 70%, 80%, 90% and 99%. This procedure and exclusion of significant parts of INDI was designed to contrast the increase in the volume of INDI data with respect to sdAB-DB an iCAN regarding data sources that were initially used to compile it. We clustered a total of 20 036 sequences (2391 from iCAN, 1452 from sdab-DB and rest from INDI) and we give the statistics from this clustering in Table 6. For each cutoff, we noted the number of clusters where we did not have any sdAB-DB nor iCAN sequences and thus calculated the number of INDI-only sequences from such groups. Table 6 demonstrates that even at the lowest sequence identity of 70%, that mostly encompasses framework differences, there are 1,453 INDI-only sequences. At higher sequence identity cutoffs (>80%) that increasingly take paratope differences into account, INDI-only sequences account for majority of all clustered sequences.

**Table 6.** Clustering contrast of INDI, sdAB-DB and iCAN

CD-HIT sequence identity cutoff	Total clusters	Clusters without sdAB-DB nor iCAN sequences	#sequences from clusters without sdAB-DB nor iCAN sequences	Clusters with sequences only from sdAB-DB	Clusters with sequences only from iCAN
70%	784	444	1453	27	11
80%	3903	2577	7493	45	57
90%	7725	5528	12 356	59	125
99%	13 285	10 041	13 456	73	251

We clustered 1452 sequences from sdAB-DB and 2391 sequences from iCAN together with three automatically-obtainable subsets of INDI: patents, structures and GenBank. Manual sequences and NGS were left out from this comparison so as not to saturate the clustering with 11 million NGS sequences and to avoid non-automatically obtained manual sequences. A total of 17 645 sequences were clustered together using CD-HIT. The columns indicate the number of clusters and the clusters and numbers of sequences without any sdAB-DB sequences as well as number of clusters with only sdAB-DB sequences.

## USAGE

We mapped the most common retrieval tasks to facilitate interaction with INDI online and offline. Through our website (http://naturalantibody.com/nanobodies) users are able to perform nanobody-specific sequence-based searches and metadata retrieval. To facilitate offline immunoinformatic analyses, we make the data available for bulk download.

### Sequence-based search

We make two nanobody-specific sequence search functions available to facilitate interaction with the data in INDI—*Variable Region Search* and *CDRH3* search. The division reflects the two-common use-cases of nanobody sequence identification. The former addresses retrieval of the entirety of the variable region. The latter addresses specific searches of the most variable portion of the nanobody responsible for most of the antigen-contacts, namely CDRH3.

Variable Region Search addresses retrieval of the entire nanobody sequences that are best matched to the query. In order to reflect the nanobody-specific nature of the search, we compare nanobody sequences using the IMGT scheme, which provides an immunoglobulin-specific framework for alignment of antibodies/nanobodies. The query sequence is IMGT-numbered and subsequently aligned to the pre-numbered nanobody sequences in INDI based on IMGT-positions. The results are sorted by the highest sequence identity over the entire variable region. The results are given in an interactive sortable table that leads to more detailed results on each hit. Users can sort the results by the entire variable region sequence identity as well as the IMGT-identity to individual CDRs.

Of the three-heavy chain CDRs, CDRH3 carries the largest number of antigenic contacts (15) and is often used as a proxy for antigenic specificity by itself. Therefore, we equipped INDI with a search facility retrieving CDRH3, disregarding the rest of the variable region. Input to CDRH3 search is the IMGT-defined sequence of the CDRH3. The sequence is then divided into *k*-mers with *k* = 4. The query k-mers are matched against these precomputed for each sequence in INDI. The hits are sorted by the number of *k*-mers in common with the query. Results are subsequently aligned using global pairwise alignment algorithm as implemented in Biopython. This allows for length-independent retrieval of sequence similar CDRH3s matches. The CDRH3 results are presented in an interactive sortable table that allows the user to browse through the results and follow links to variable sequences and their associated metadata.

### Text search

Nanobody sequences in INDI are associated with rich textual annotations revealing among others biological targets, origins and purpose of the study of the molecules. Metadata fields are heterogenous across the sources and within them. For instance, metadata associated with structures will contain specific crystallographic parameters not present in other databases. In GenBank, information about the target of a nanobody can be contained within the description of the accession or the individual translations as there is no standardized way to report such information. Earlier endeavors at capturing antibody/nanobody target information relied on large-scale manual curation (18,19,35). Given that INDI encompasses three automatic components, regular manual annotation of all the entries is challenging. The great diversity in text representations poses a challenge in document retrieval.

To address the problem of information retrieval across the five diverse sources, we implemented a text index created on all the metadata fields in all the databases. User is asked to provide the keywords of interest and INDI will retrieve the accessions best matching the results. Users can specify possible targets of nanobodies that are reported as part of the depositions (e.g. protein name VEGF) as well as individual accession numbers (e.g. PDB accession 7JOO).

Results are displayed as an interactive table listing the accessions, source databases and text fields. Users can sort through the results and display the details of matching text entries. The details of text entries are displayed together with nanobody sequences linked to the accession.

### Bulk download

To supplement our web-based retrieval we make the data available for offline use as well. The data are available as an extract of the two pillars of our data model – sequences and metadata separately. The sequence-extract contains the V-region sequences of nanobodies we identify. Each sequence entry is linked to the metadata fields contained within the meta-extract. Metadata fields are sorted by one of the five databases. All data is available through the main INDI website available at http://naturalantibody.com/nanobodies. Additionally, the August 2021 snapshot of INDI was deposited on FigShare to assure persistence of the data (https://figshare.com/projects/INDI_-_Integrated_Nanobody_Database_for_Immunoinformatics/122022) together with inclusion of a supplementary Excel spreadsheet associated containing all the data but NGS (due to size).

## DISCUSSION

Delivering an antibody drug to clinical use requires a big investment of time and resources with a high likelihood of failure at the clinical trials stage. Novel formats such as nanobodies with favorable biophysical properties, offer opportunities to mitigate certain drug discovery risks (5,36). Innovative approaches for targeted delivery of nanobody-based therapeutics are being pursued currently (37). Besides the molecular therapies (37), nanobodies are being used in the development of several cellular therapies (37–41). Developing nanobody therapies using traditional lab-based approaches still carries an overhead of many years of experimentation before they reach the clinic. Computational approaches could accelerate this process, delivering life-saving therapeutics much faster.

Though still in its infancy, bioinformatic methods addressing issues of therapeutic nanobody design are being developed. Computational nanobody approaches can provide insight in developing reliable structural modeling methods (42), design of phage display libraries (43) or computational design of novel nanobodies (16). Parallels between antibodies and nanobodies allow certain protocols to transfer information between the two types of molecules. For instance, though nanobody-specific structural modeling pipelines exist (42), it is possible to obtain reliable models of nanobody structures employing antibody protocols (44).

Despite certain parallels between nanobodies and antibodies, contrast between the binding sites of the two reveals certain differences (15). Though there is an overlap between nanobody and antibody epitopes, either is capable of binding molecular surfaces that the other might find challenging (45). Deconvoluting such nuanced distinctions is required to understand the binding mode of nanobodies ultimately leading to reliable computational nanobody design protocols (16). Any computational efforts however require sound access to nanobody sequence data.

To address this need, here we developed INDI, a database integrating nanobody sequences, structures and their associated metadata in the public domain. Automatic updates from the heterogeneous sources make it possible to keep up with the accelerating pace of deposition in the public domain. Heterogeneity of data in INDI allows nanobody researchers to obtain an accurate picture of the current state of knowledge of nanobody sequence, structure and function. Such knowledge can then accelerate the development of analytical frameworks (14,15), structural modeling (42), *de novo* nanobody design protocols (16) and as a basis for deep-learning models addressing nanobody design (17). Altogether we hope that INDI will form a solid data foundation to develop nanobody-specific computational methods that will accelerate development of novel therapeutics in this format.

## Supplementary Material

gkab1021_Supplemental_File
